# Continuous Gums

**Published:** 1855-10

**Authors:** 


### ARTICLE V.

*Continuous Gums. Report of* DR. LOCKE, of Cincinnati.

CINCINNATI, *May* 12, 1855.

GENTLEMEN: Previous to entering upon the discussion of the characteristics of the several specimens of porcelain, termed "Base" for artificial gums, I will draw your attention to some of the terms which it will be necessary to make use of, as an explanation of such terms will better enable us to express ourselves and prevent useless repetition.

It is desirable upon entering into the discussion of any objects to make ourselves acquainted with that substance, therefore, we will make the following quotation from Knapp's Chemical Technology, vol. ii, page 219:

"*True Porcelain.*—Nature of the Mass.—The clay wares which are known under this name, and which are manufactured in China, and in all European countries (with the exception of England) are composed of two essentially different constituents, the one of which is an infusible, plastic white clay, called, kaolin or China clay; the other, an infusible, not plastic, material, the so-called flux, the latter, is almost universally composed of feldspar, with the addition of quartz, chalk and gypsum.

"Kaolin alone, would afford a porous, opaque body; the flux, however, softens in the heat of the porcelain kiln, and penetrates as a nitreous matter, the whole body of the clay, completely filling all the pores, and covering all the surface, it binds the whole together as a dense impenetrable mass. The translucency of this material is, therefore, due to the clay body being saturated as it were with a mass of glass; as transparent paper is permeated with oil. Under the microscope, the two ingredients can be clearly distinguished, separate from each other, and the milky mass appears as a transparent ground mixed with an opaque ingredient, which, according to Ehrenberg's observations, consists of articulated threads (i. e. composed of globules arranged in a linear direction, one on the other) or little rods, which are interwoven and cross each other in all directions."

A proximate analysis, is such an analysis as would exhibit the articles, in common parlance, which compose the substance, thus, brick and mortar are the proximate portions of a brick wall.

Ultimate analysis consists in giving all the principles in an isolated condition, thus, the ultimate analysis of the brick wall would be, alumina, iron, oxygen, silica, &c., which enter into the composition of the bricks and lime, silica, &c., which forms the mortar.

"Base," is not here used in its chemical sense, but as a technical term, in dentistry, signifying the substance used for artificial gums, the coloring being a separate substance which is afterwards placed upon the base.

*Report.*—M. W. S. Kendall assisted me in preparing the "Base" upon which I experimented, they were not prepared by myself, on account of the limited time which I had for making my examination, nevertheless, they were prepared under my superintendence whilst employed at other parts of the examination.

The formulas used, were taken from the following sources: That spoken of as "according to De La Barre's formula" was taken from Fitch's Dental Surgery, second edition, page 471, (published 1835.) It read as follows:

| | Parts. |
|---|---|
| Porcelain paste, . . . | 70 |
| Calcined gypsum, . . . | 10 |
| White sand, . . . . | $\frac{1}{20}$ the mass. |

What oxyd you please, 50 grammes, by kiagrams.

The Allen formula reads as follows:

| | |
|---|---|
| Silex, . . . . | 2 oz. |
| Flint glass, . . . | 2 oz. |
| Borax, . . . . | 1 oz. |
| Asbestos, . . . . | 2 drs. |
| Wedgewood, . . . | 1½ oz. |
| Feldspar, . . . . | 2 drs. |
| Kaolin, . . . . | 1 drm. |

This receipt was taken from a printed paper entitled "The Schedule," referred to in these *Letters Patent*, and making part of the same.

Dr. Hunter's formula is the one published in vol. vi, No. xii of the N. York Dental Record, 1852, and reads thus:

*Granulated Body.*—Take any hard tooth material (I use the following formula: spar 8 oz., silex 1½ oz., kaolin ½ oz.) and fuse completely, &c.

Flux silica 8 oz., calcined borax 4 oz., caustic potash 1 oz., grind, &c.

*Base.*—Take flux 1 oz., asbestos 2 oz., grind together, very fine, completely intermixing. Add granulated body 1½ oz. and mix with a spatula, &c.

It is a well known and admitted fact, that potters change frequently the formula from which they prepare their cases.

This results from the different quantitative ultimate analysis of different lots of the same proximate materials, they being guided more by the ultimate analysis of the porcelain when finished, than by the proportion of the proximate principles, but, nevertheless, these proximate principles have some slight influence upon the case. I will quote from the formulas of the celebrated manufactories of Sevres in France in support of the former portion of this statement.

"The mixture must consist in a hundred parts of

58.0 silica,
34.5 alumina,
4.5 lime, and
3.0 potash.

TABLE I.

| Mixture of 1839. Composed of | Contains Silica. | Alumina. | Lime. | Potash. |
|---|---|---|---|---|
| 73 Decantea kaolin, ........... | 30.69 | 30.66 | 0.73 | 1.75 |
| 24 Feldspatic sand, .......... | 19.27 | 3.40 | | 1.27 |
| 6.5 Chalk, .................... | | | 3.77 | |
| | 57.96 | 34.06 | 4.50 | 3.02 |

TABLE II.

| Mixture of 1849. Composed of | Contains Silica. | Alumina. | Lime. | Potash. |
|---|---|---|---|---|
| 48 Decantea kaolin, ........... | 30 | 17 | 0.05 | 0.96 |
| 48 Feldspatic sand, ............ | 28 | 17 | 0.53 | 2.01 |
| 7.2 Chalk, | | | 4.00 | |
| | 58 | 34 | 4.58 | 2.97 |

By referring to this table it is immediately evident that the receipt for compounding the clay was remarkably different in the years 1839 and 1849; it will also be observed that the specimens of kaolin and feldspar respectively were remarkably different, thereby showing that they are not definite chemical compounds, but a mechanical mixture of the ingredients which admit of being varied; with all these changes we notice an almost identical resemblance of the ultimate analysis of the two formulas to the desired proportions.

This clearly proves that the proximate analysis may indicate a total want of identity between two pieces of ware, which identity would be fully established if we regarded the ultimate analysis.

I have reduced the three formulas which at present occupy our attention down to formulas of per centage, thus:

TABLE III.
Dr. Allen's Formula.

| | |
|---|---|
| Silica, ....... | 28.070 |
| White glass, | 28.070 |
| Borax, ...... | 14.035 |
| Wedgewood, | 21.053 |
| Asbestos, ..... | 3.509 |
| Spur ......... | 3.509 |
| Kaolin ....... | 1.754 |

TABLE IV.
Dr. Hunter's Formula.

| | | |
|---|---|---|
| 22.222-\|- Flux, | Potassa, | 1.709 |
| | Silica | 13.675 |
| | Col. borax | 6.834 |
| 44.444-\|- | Asbestos, | 44.444-\|- |
| 33.333-\|- Granulated body, | Spar, | 20.000 |
| | Silica, | 10.000 |
| | Kaolin, | 3.333-\|- |

TABLE V.
Delabarre's Formula.

| | |
|---|---|
| Porcelain paste, | 72.464 |
| Calcined gypsum, | 10.352 |
| White sand, ...... | 4,141 |
| Oxyd, ............ | 13.043 |

From these foregoing tables I have arranged the following tables, which exhibit the proportion of proximate substances in each base and ultimate analysis of each base and proximate constituent of base.

Tables exhibiting the proximate and ultimate analysis of the several "Gum Bases."

| | The proximate constituents taken from the formula. | Silica. | Alumina. | Lime. | Magnesia. | Oxyd of Iron. | Oxyd of Lead. | Potash or Alkali. | Soda. | Sulphuric Acid. | Boracic Acid. | Foreign matter, loss, &c. | Temperature at which the base vitrifies. |
|---|---|---|---|---|---|---|---|---|---|---|---|---|---|
| TABLE VI. *According to Delabarre's Formula.* | 72.464 Porcelain paste, | 42.029 | 25.000 | 3.261 | | | | 2.174 | | | | | 3761° |
| | 10.352 Calcined gypsum, | | | 4.291 | | | | | | 6.061 | | | |
| | 4.141 White sand, | 4.141 | | | | | | | | | | | |
| | 13.043 Red lead, | | | | | | 13.043 | | | | | | |
| | Total, | 46.169 | 25.000 | 7.552 | | | 13.043 | 2.174 | | 6.061 | | | |
| TABLE VII. *According to Dr. Allen's Formula.* | 28.070 Silica, | 28.070 | | | | | | | | | | | 2264° |
| | 28.070 White glass, | 12.435 | | | | | 12.085 | 3.297 | | | | 0.253 | |
| | 14.035 Calcined borax, | | | | | | | | 4.491 | | 9.544 | | |
| | 21.053 Wedgewood, | 13.998 | 5.474 | 0.219 | 0.032 | 1.288 | | 0.042 | | | | | |
| | 3.509 Asbestos, | 2.116 | 0.009 | 0.479 | 0.850 | 0.005 | | | | | | 0.050 | |
| | 3.509 Feldspar, | 2.342 | 0.614 | 0.044 | | 0.026 | | 0.421 | | | | 0.062 | |
| | 1.754 Kaolin, | 0.995 | 0.690 | 0.001 | | | | 0.042 | | | | 0.011 | |
| | Total, | 59.956 | 6.787 | 0.743 | 0.882 | 1.319 | 12.085 | 3.802 | 4.491 | | 9.544 | 0.376 | |
| TABLE VIII. *According to Dr. Hunter's Formula.* | 22.22† Flux. { 1.709 Potassa, | | | | | | | 1.709 | | | | | 2264° |
| | 13.675 Silica, | 13.675 | | | | | | | | | | | |
| | 6.838 Cal. borax, | | | | | | | | 2.188 | | 4.650 | | |
| | 4.444 Asbestos, | 26.804 | 0.116 | 6.071 | 10.769 | 0.067 | | | | | | 0.618 | |
| | 33.33† Gran. Body. { 20.0 Spar, | 13.350 | 3.500 | 0.250 | | 0.150 | | 24.000 | | | | | |
| | 10.0 Silica, | 10.00 | | | | | | | | | | | |
| | 33.33† Kaolin, | 1.892 | 1.313 | 0.021 | | | | 0.075 | | | | 0.032 | |
| | Total, | 65.721 | 4.929 | 6.342 | 10.769 | 0.217 | | 4.184 | 2.188 | | 4.650 | 0.650 | |
| TABLE IX. *Ultimate Analyses of Bases, according to Delabarre, Hunter and Allen.* | Delabarre, | 46,169 | 25.000 | 7.552 | | | 13.043 | 2.174 | | 6.061 | | | |
| | Allen, | 59.956 | 6.787 | 0.743 | 0.882 | 1.319 | 12.085 | 3.802 | 4.491 | | 9.544 | 0.376 | 2264° |
| | Hunter, | 65.721 | 4.929 | 6.342 | 10.769 | 0.217 | | 4.184 | 2.188 | | 4.650 | 0.650 | |

But few remarks are needed upon these tables. They are not composed from actual analysis, but by calculation from the proportion of the ingredients whose analysis have been collected from the best authorities, (see appendix,) but we will speak more fully of this subject hereafter.

The following table exhibits the per centage of linear contraction of platinum and the three specimens of "Base:" In cooling, from 1645° Fahrenheit to 60° Fahrenheit, the former being the temperature at which the specimens prepared, according to Hunter's and Allen's formula, become solid in cooling, after having been heated to vitrification or softening.

TABLE X.

| The formula by which the base was prepared. | Per ct. of linear contraction. | From | To | Number of degrees through which it cooled. |
|---|---|---|---|---|
| Delabarre's baked only to 2264° | 0.0461 | 1645° | 60° | 1585° |
| " " " to 3761° | 0.0486 | " | " | " |
| Dr. Hunter's " to 2264° | 0.0774 | " | " | " |
| Dr. Allen's " to 2264° | 0.0767 | " | " | " |
| Platinum, (by calculation,) | 0.0790 | | | |

Having found the per centage of contraction, I was desirous of ascertaining the amount which a platina plate would be distorted by the difference of contraction of the plate and the gum "Base" upon it. Having measured ten jaws from second molar to second molar, I found the mean to be 1.77 inches, which size jaw the calculations were made upon and presented in

TABLE XI.

| From whose formula prepared. | Delabarre not verified but baked to 2264° | Delabarre fused at 3761° | Allen's Formula. | Hunter's Formula. |
|---|---|---|---|---|
| Contraction of base from second molar towards second molar, | inches. 0.00815 | 0.00861 | 0.01278 | 0.01329 |
| Contraction of platina plate in same direction, | 0.014 | 0.014 | 0.014 | 0.014 |
| Difference of contraction of base and platina plate, | 0.00585 | 0.00539 | 0.00122 | 0.00071 |
| Temperature from which they cooled, | 1645° | 1645° | 1645° | 1645° |
| Temperature to which they cooled, | 60° | 60° | 60° | 60° |

The composition, according to Delabarre, was quite plastic when moist, on account of the amount of alumina which it contained; when heated, it shrank from the plate, this was, also, owing to the moist or hydrated alumina, which is remarkable for its great contraction, a secondary effect of the contraction was the leaving of large pores, and when the heat was carried to that height that the portion which vitrifies arrived at that state, the pores were too large to draw in the vitreous mass, hence it was left a porous mass, the heat required for vitrifaction was 3761° Fahrenheit, being about 300° above the temperature required for the melting of iron.

The general character of the specimens of Drs. Allen and Hunter's compound was so close that the description of one will answer for both. They were less plastic than the preceding, (Delabarre's,) they were not of the same shade of color, and required 2264° (a little above the melting of gold,) to vitrify them. At two different times I made a specimen of each, in the first experiment after vitrification, the specimen, according to Dr. Allen's formula, was the whitest, but in the second experiment the reverse was the case.

The temperatures in these experiments were taken by Daniell's pyrometer, for the lower temperatures, using an iron rod, which was substituted by a platina rod, for the highest temperatures.

Although somewhat out of place, I will here remark, that it would have been but natural and better to have made an actual analysis of the bases upon which the experiments of contraction, &c. were made. The reason of it not having been done was simply that I was called upon at a time too shortly prior to that at which the report was to be delivered.

*Conclusions.*—The question naturally arises, if the alumina produces such deleterious effects in Delabarre's compound, why do they succeed in making china containing more alumina; the reasons are, that any vessel composed of china paste, shrinks proportionally in every part, thereby, becoming only smaller without distortion, as it is not connected to any other substance which does not shrink permanently, the heat can then be carried to such an extent as to render the vitreous mass sufficiently fluid, to be taken up into the pores, but, were the ordinary porcelain teeth present at such a heating, they would be fused down into a mass, this precludes the practicability of Delabarre's method; there are two other difficulties to be encountered; if we had teeth sufficiently hard, the great heat required would preclude the method, but the difference of contraction between platinum and this compound, is such as to distort the work, the difference being even greater than shown in Table XI, as the temperature at which the base would commence to assume a hard character would be considerable above 1645°.

In our trial of Delabarre, we gave it an advantage by using a porcelain paste which is more easily vitrified than those of France, which are the pastes he refers to, as he directs the person to procure it from the porcelain works. In my Table V, I used the analysis of the Sevres porcelain paste, as it is the best French article.

The difference between the Delabarre and Drs. Hunter and Allen's (for we will consider them collectively,) consists in their containing so small a quantity of alumina, that in drying they do not shrink from, and, therefore, not fit to the platina plate, nor does it leave the mass so porous and render it so difficult for the vitreous matter to permeate it. They (the Allen and Hunter compounds) only require a practical heat, their rate of contraction approaches closely to that of platinum, (Table X,) both of which cases Delabarre's compound does not fulfil.

We have previously spoken of Drs. Allen and Hunter's compounds, collectively; our reason for so doing are these: they agree so closely in physical character, they vitrify at the same temperature, and approach one another remarkably close in contraction, &c. We will look for a moment to the principle upon which these bases are constructed. Some granular refractive substance, not effected by ordinary heat, is held into position by the means of hydrated alumina as a paste or glue, and disseminated through this mass is another or other substances which are vitrified at a medium temperature, and solders together, the less vitrifiable particles in the form which they have been slightly held by the alumina and by referring to the ultimate analysis, (Tables VII and VIII,) you will observe nearly the same per cent. of alumina which is used as paste. The quantity of silica also approach one another, were Bohemian glass to be used in Dr. Allen's formula, then the quantity of silica would be augmented, and no oxyd of lead would be present, they would then approach each other still more closely. The large quantity of lime and magnesia in Table VIII, is owing to so much asbestos being introduced for the article which is to be soldered by the vitreous mass. This part of the mass in Dr. Allen's formula is the wedgewood, (which resembles asbestos, see analysis in appendix,) asbestos, alumina and part of the silica. The quantity of matter, usually termed flux, for uniting with the silica, &c., to form the vitreous portion is about equal in the two. In Dr. Allen's formula, we have from the glass, ox. of lead 12.08, and potash 3.30, calcined borax 14.03, and 3.50 of feldspar, making 32.9 per cent. Dr. Hunter's formula contains potassa 1.7, calcined borax 6.84, and feldspar 20.00, total 28.5 per cent., as in the two formulas, there are different proportions of various fluxes; we are not enabled to arrive at their exact arithmetic value for forming vitreous mass.

*Synopsis.*—In our opinion we have shown that Delabarre's formula is impracticable, Drs. Hunter and Allen's are identical in physical character and properties, their formation involves precisely the same principle, the only difference being a partial substitution of one article for another.

